# Supplementary material for: Hepatic ceramide synthesis links systemic inflammation to organelle dysfunction in cancer
Source: bioRxiv. 2025 Oct 16:2025.10.01.679814. Preprint. [Version 2] doi: 10.1101/2025.10.01.679814 (PMC12621733; doi:10.1101/2025.10.01.679814)
Supplement: 1 [file NIHPP2025.10.01.679814V2-supplement-1.pdf]

bioRxiv preprint doi: https://doi.org/10.1101/2025.10.01.679814; this version posted October 16, 2025. The copyright holder for this preprint (which was not certified by peer review) is the author/funder, who has granted bioRxiv a license to display the preprint in perpetuity. It is made available under aCC-BY 4.0 International license.

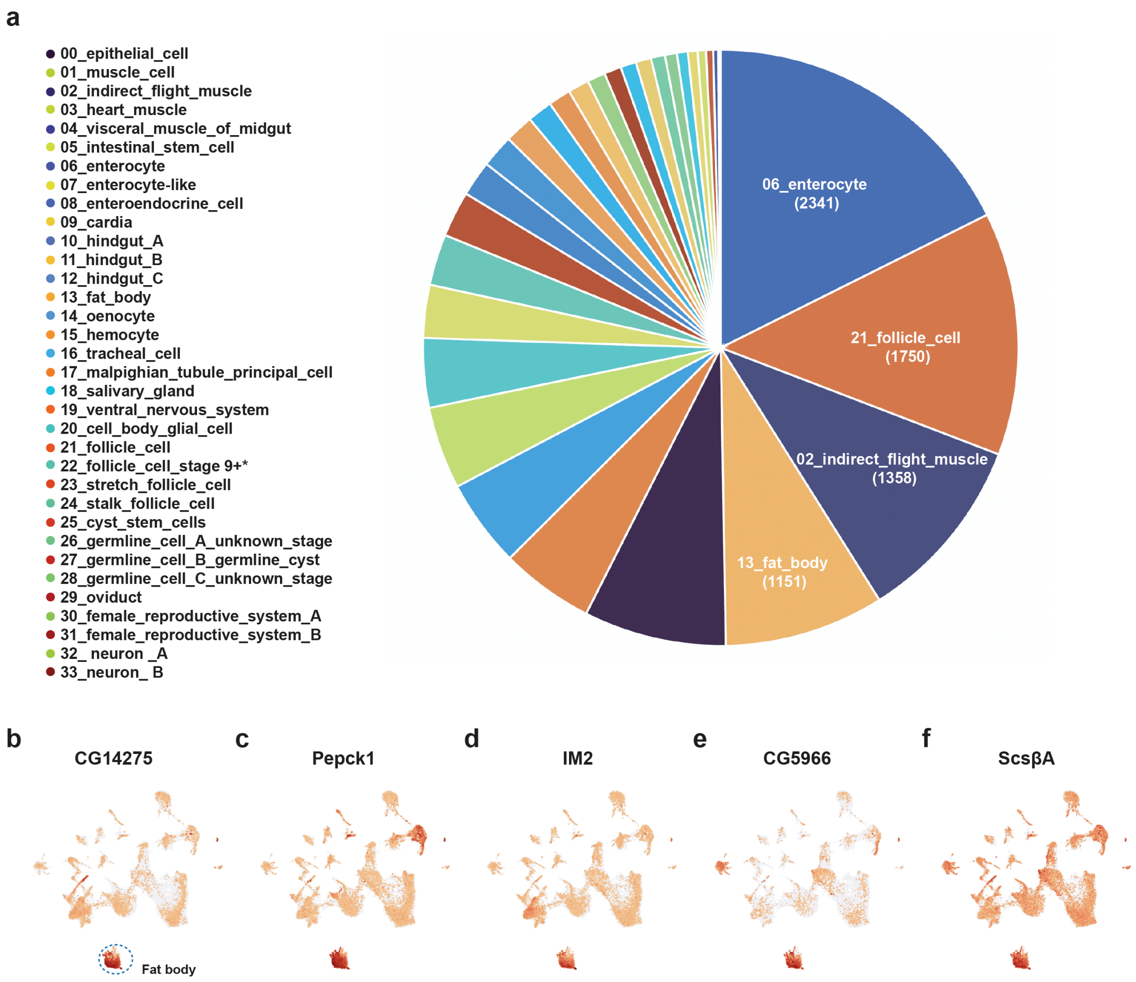


987

bioRxiv preprint doi: https://doi.org/10.1101/2025.10.01.679814; this version posted October 16, 2025. The copyright holder for this preprint (which was not certified by peer review) is the author/funder, who has granted bioRxiv a license to display the preprint in perpetuity. It is made available under aCC-BY 4.0 International license.

**Figure S1, related to Figure 1.**

**a**, Distribution of progression associated genes across cell clusters. **b**-**f**, Expression of CG14275 (**b**), Pepck1 (**c**), IM2 (**d**), CG5966 (**e**), ScsbetaA (**f**) across cell clusters, data retried from snRNA-seq.

bioRxiv preprint doi: https://doi.org/10.1101/2025.10.01.679814; this version posted October 16, 2025. The copyright holder for this preprint (which was not certified by peer review) is the author/funder, who has granted bioRxiv a license to display the preprint in perpetuity. It is made available under aCC-BY 4.0 International license.

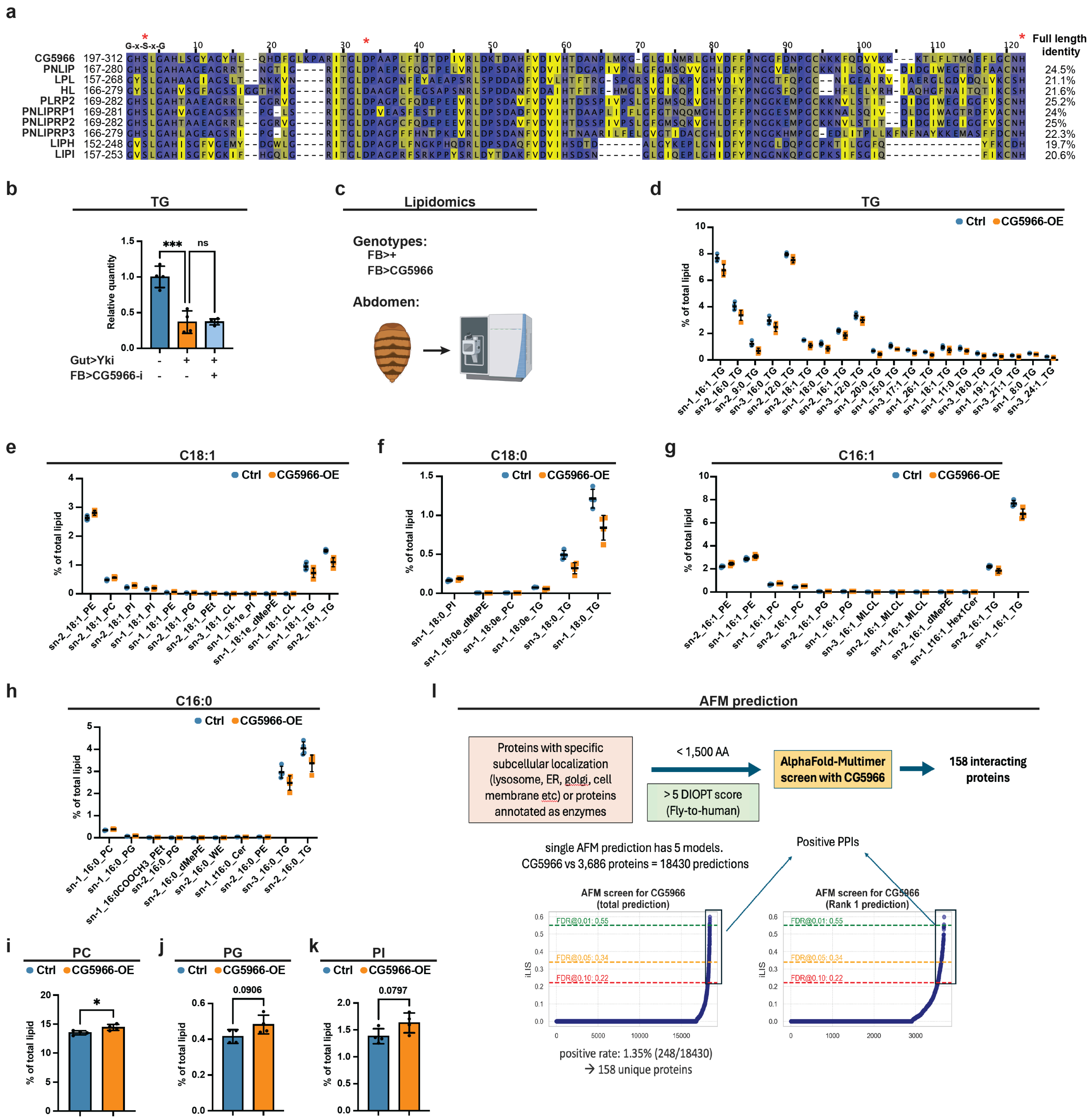

bioRxiv preprint doi: https://doi.org/10.1101/2025.10.01.679814; this version posted October 16, 2025. The copyright holder for this preprint (which was not certified by peer review) is the author/funder, who has granted bioRxiv a license to display the preprint in perpetuity. It is made available under aCC-BY 4.0 International license.

**Figure S2, related to Figure 2.**

**a**, Amino acid sequence alignments showing similarity of human lipases to *CG5966*. **b**, Whole-body TG levels of Yki flies with or without fat-body *CG5966* depletion at day 6 (n = 4). **c**, Lipidomics analysis setting. **d**-**h**, Proportions of significantly changed abdomen TG species (**d**), and lipid species containing C18:1 (**e**), C18:0 (**f**), C16:1 (**g**), or C16:0 (**h**) acyl chains in flies with or without fat-body *CG5966* overexpression (n=4), data were retrieved from lipidomics analysis. **i**-**k**, Proportions of abdomen PC (**i**), PG (**j**), PI (**k**) of flies with or without fat-body CG5966 overexpression (n=4), data were retrieved from lipidomics analysis. **l**, AFM analysis setting. *p < 0.05, **p < 0.01, ***p < 0.001, ****p < 0.0001, ns indicates not significant. Error bars indicate SDs. n indicates the number of biological replicates in each experiment.

bioRxiv preprint doi: https://doi.org/10.1101/2025.10.01.679814; this version posted October 16, 2025. The copyright holder for this preprint (which was not certified by peer review) is the author/funder, who has granted bioRxiv a license to display the preprint in perpetuity. It is made available under aCC-BY 4.0 International license.

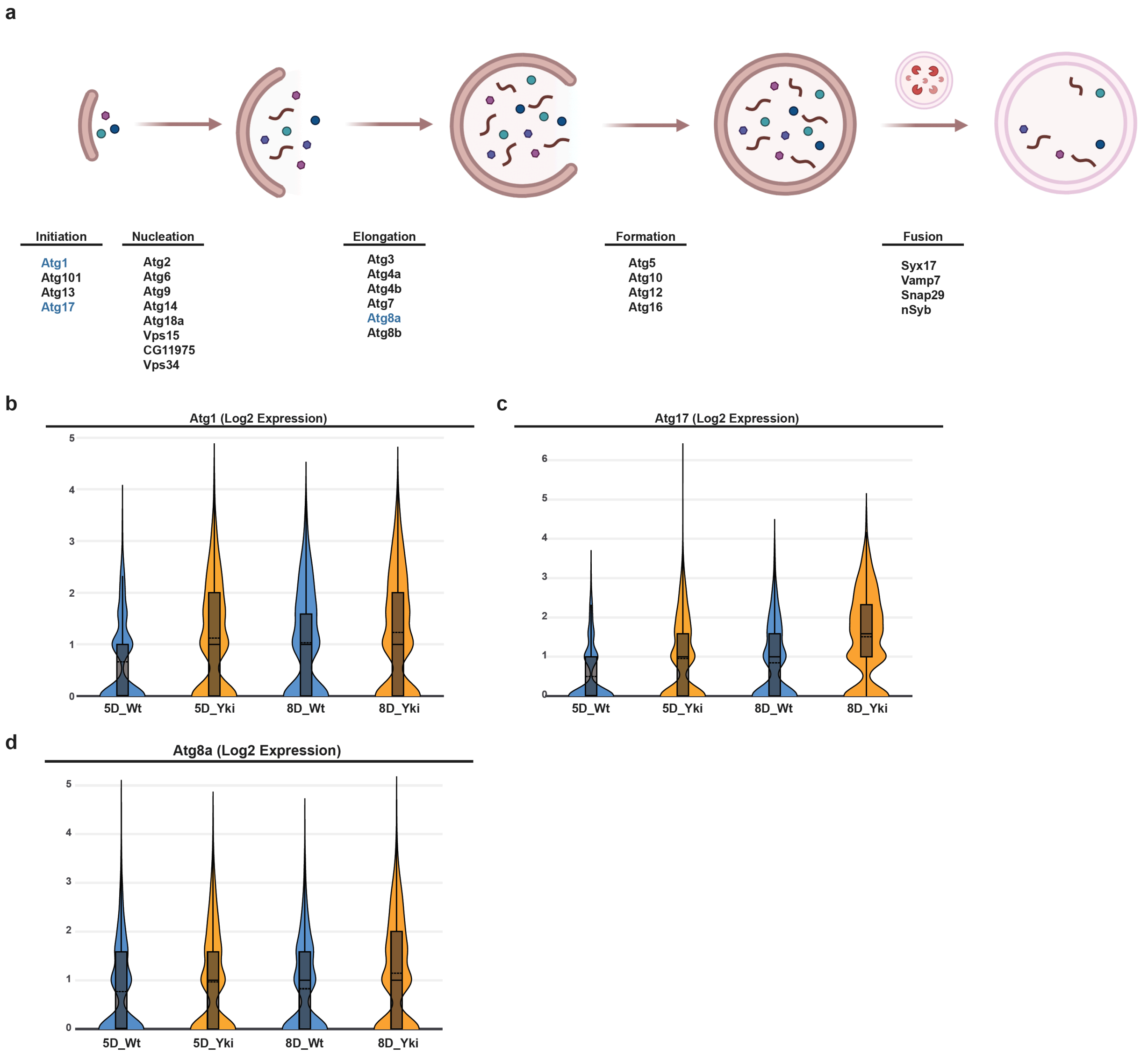


1004

bioRxiv preprint doi: https://doi.org/10.1101/2025.10.01.679814; this version posted October 16, 2025. The copyright holder for this preprint (which was not certified by peer review) is the author/funder, who has granted bioRxiv a license to display the preprint in perpetuity. It is made available under aCC-BY 4.0 International license.

**Figure S3, related to Figure 3.**

**a**, A schematic of autophagy in *Drosophila*, genes upregulated in Yki are labeled in blue, other genes are not significantly changed. **b-d**, Log2 expression of *Atg1* (**b**), *Atg17* (**c**), and *Atg8a* (**d**) in day 5 and day 8 control and Yki flies, data were retrieved from snRNAseq analysis.

bioRxiv preprint doi: https://doi.org/10.1101/2025.10.01.679814; this version posted October 16, 2025. The copyright holder for this preprint (which was not certified by peer review) is the author/funder, who has granted bioRxiv a license to display the preprint in perpetuity. It is made available under aCC-BY 4.0 International license.

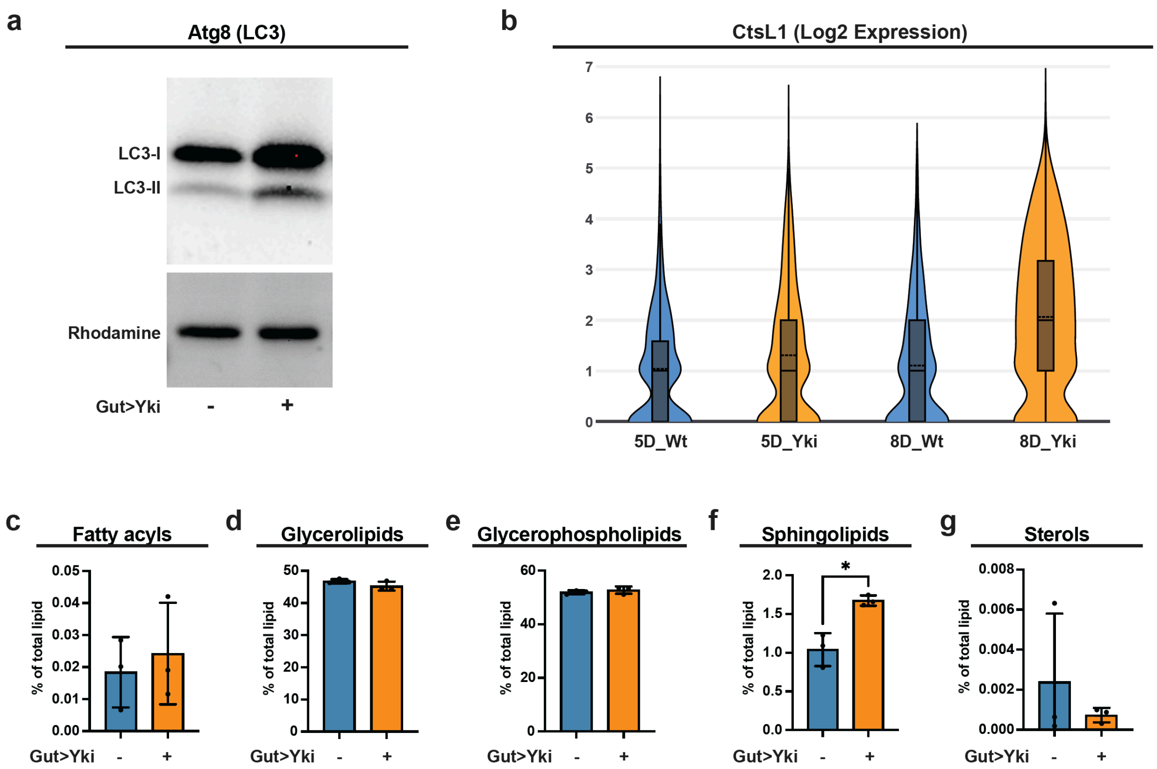

bioRxiv preprint doi: https://doi.org/10.1101/2025.10.01.679814; this version posted October 16, 2025. The copyright holder for this preprint (which was not certified by peer review) is the author/funder, who has granted bioRxiv a license to display the preprint in perpetuity. It is made available under aCC-BY 4.0 International license.

**Figure S4, related to Figure 4.**

**a**, Western blot of Atg8 (LC3) of abdomen samples from control and Yki flies. Rhodamine was used as loading control. **b**, Log2 expression of *CtsL1* in day 5 and day 8 control and Yki flies, data were retrieved from snRNAseq analysis. **c**-**g**, Whole-body proportions of lipid subgroups fatty acyls (**c**), glycerolipids (**d**), glycerophospholipids (**e**), sphingolipids (**f**), and sterols (**g**) in control and Yki flies, data were retrieved from lipidomics analysis.

bioRxiv preprint doi: https://doi.org/10.1101/2025.10.01.679814; this version posted October 16, 2025. The copyright holder for this preprint (which was not certified by peer review) is the author/funder, who has granted bioRxiv a license to display the preprint in perpetuity. It is made available under aCC-BY 4.0 International license.

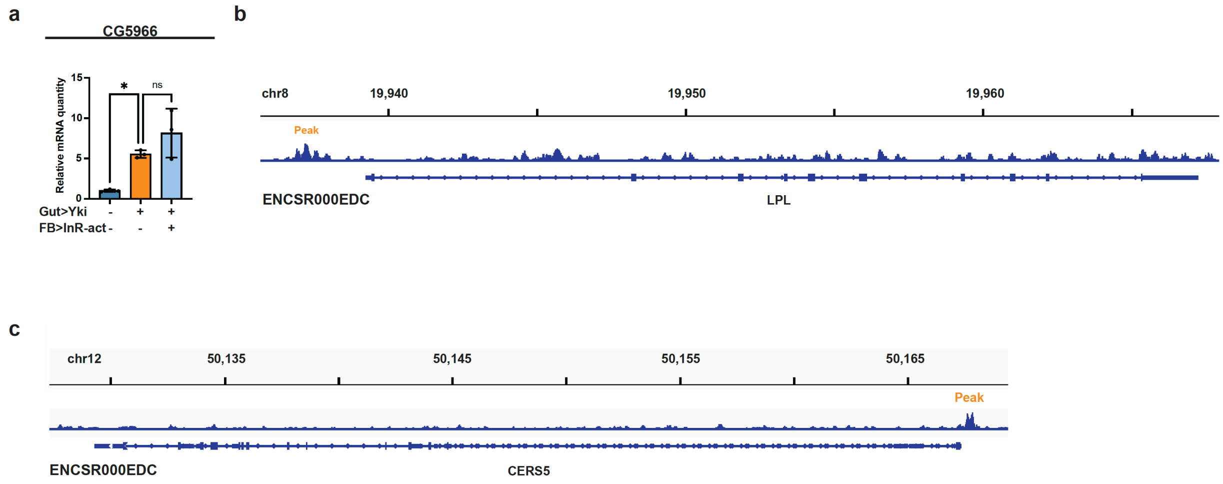


1017

bioRxiv preprint doi: https://doi.org/10.1101/2025.10.01.679814; this version posted October 16, 2025. The copyright holder for this preprint (which was not certified by peer review) is the author/funder, who has granted bioRxiv a license to display the preprint in perpetuity. It is made available under aCC-BY 4.0 International license.

**Figure S5, related to Figure 5.**

**a**, *CG5966* mRNA expression in the fat body of control, Yki flies, and Yki flies with fat-body *InR-act* (active form of InR) expression at day 6 (n = 3). *$p < 0.05$, **$p < 0.01$, ***$p < 0.001$, ****$p < 0.0001$, ns indicates not significant. Error bars indicate SDs. n indicates biological replicates. **b**,**c**, Data from the ChIP-seq data (ENCSR000EDC) indicating enrichment of STAT3 binding at the *LPL* (**b**) and *CERS5* (**c**) promoter and gene regions.

bioRxiv preprint doi: https://doi.org/10.1101/2025.10.01.679814; this version posted October 16, 2025. The copyright holder for this preprint (which was not certified by peer review) is the author/funder, who has granted bioRxiv a license to display the preprint in perpetuity. It is made available under aCC-BY 4.0 International license.

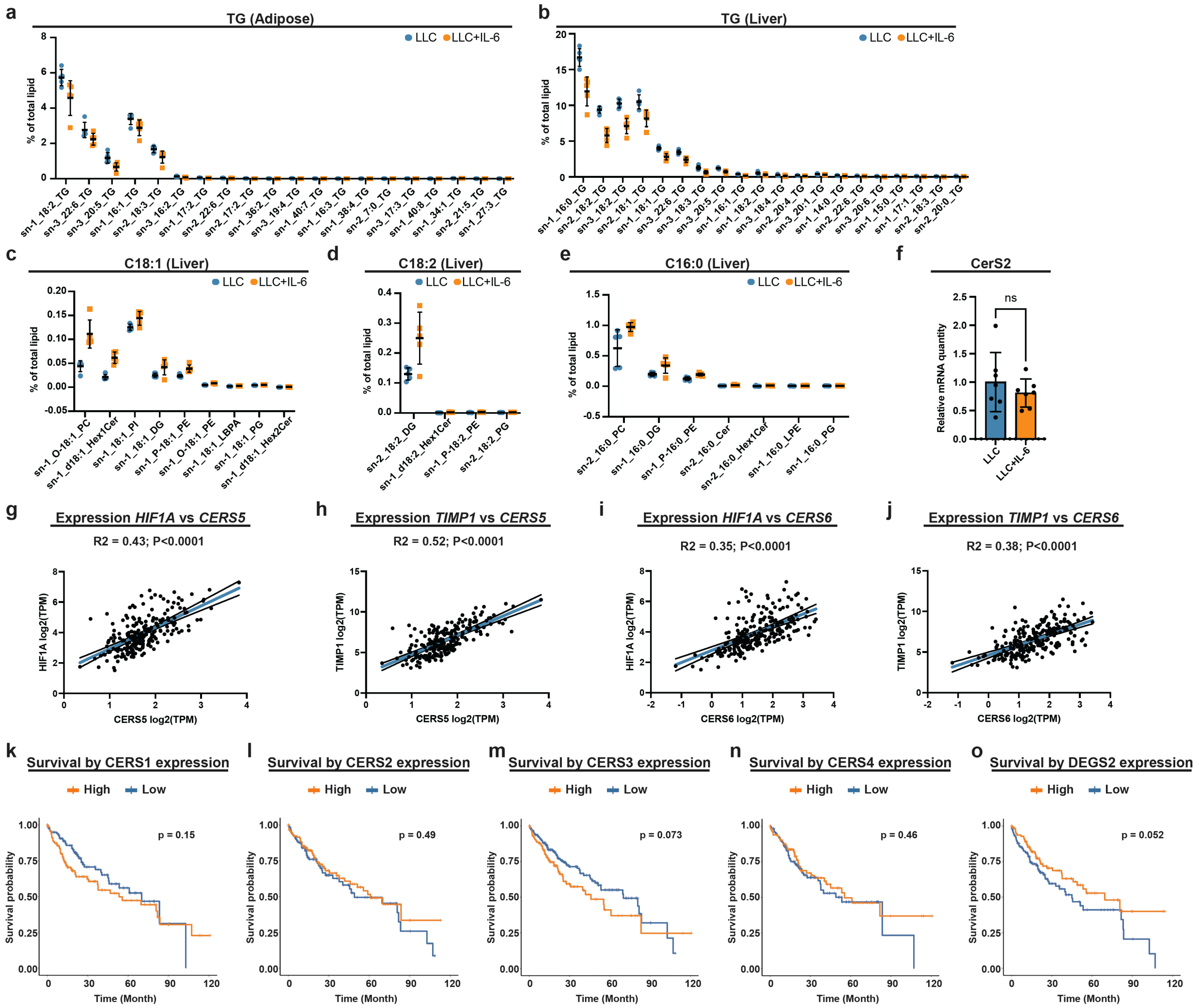


1024

bioRxiv preprint doi: https://doi.org/10.1101/2025.10.01.679814; this version posted October 16, 2025. The copyright holder for this preprint (which was not certified by peer review) is the author/funder, who has granted bioRxiv a license to display the preprint in perpetuity. It is made available under aCC-BY 4.0 International license.

**Figure S6. related to Figure 6.**

**a**,**b**, Proportions of significantly changed adipose (**a**) and liver (**b**) TG levels of B6 mice injected with LLC cells with or without IL-6 expression (n=5). **c**-**e**, Proportions of significantly changed lipid species containing C18:1 (**c**), C18:2 (**d**), or C16:0 (**e**) acyl chains of B6 mice injected with LLC cells with or without IL-6 expression in liver (n=5). **f**, qRT–PCR of *CerS2* mRNA in liver of B6 mice injected with LLC cells with or without IL-6 expression (n = 7). **g**-**j**, Correlation plots showing the positive relationship between expression of *HIF1A* and *CerS5* (**g**), *TIMP1* and *CerS5* (**h**), *HIF1A* and *CerS6* (**i**), *TIMP1* and *CerS6* (**j**) in non-diseased liver samples of 226 participants in the GTEx Project. TPM, transcript per million. **k**-**o**, Kaplan–Meier survival curves displaying the estimated survival probabilities of people with hepatocellular carcinoma with low (bottom third) and high (top third) hepatic expression of *CERS1* (**k**), *CERS2* (**l**), *CERS3* (**m**), *CERS4* (**n**), and *DEGS2* (**o**). *$p < 0.05$, **$p < 0.01$, ***$p < 0.001$, ****$p < 0.0001$, ns indicates not significant. Error bars indicate SDs. n indicates the number of biological replicates in each experiment.
